# Individual, family, and community factors related to loneliness in mothers raising children less than 3 years of age: a cross-sectional study

**DOI:** 10.1186/s12905-021-01365-7

**Published:** 2021-05-31

**Authors:** Azusa Arimoto, Etsuko Tadaka

**Affiliations:** 1 Department of Community Health Nursing, Graduate School of Medicine, Yokohama City University, 3-9 Fukuura, Kanazawa-ku, Yokohama, 236-0004 Japan; 2 Department of Community and Public Health Nursing, Graduate School of Health Sciences and Faculty of Medicine, Hokkaido University, N12-W5, Kitaku, Sapporo, 060-0812 Japan

**Keywords:** Loneliness, Social isolation, Maternal and child health, Mother, Infant, Toddler, Community, Factors

## Abstract

**Background:**

Loneliness in mothers raising children under 3 years of age is a major challenge. The purpose of this study was to identify the individual, family, and community factors associated with loneliness among mothers raising children under 3 years of age with social isolation as a mediator.

**Methods:**

A cross-sectional survey was conducted using anonymous self-administered questionnaires. The target population was all 649 mothers of children under 3 years of age visiting a public health center in Yokohama City and eligible for child health examinations between November 2019 and February 2020. The study measures included loneliness (10-item version of the UCLA Loneliness Scale), social isolation (Lubben Social Network Scale [LSNS-6]), demographic data, individual factors, family factors, and community factors from an ecological systems model. Social isolation was classified based on the LSNS-6 cutoff points. Multiple regression analysis was conducted to examine the association between loneliness and individual, family, and community factors with social isolation as a mediator.

**Results:**

A total of 531 participants (81.8% response rate) responded, and 492 (75.8% valid response rate) were included in the analysis. Loneliness was significantly higher in the isolated group (n = 171, 34.8%) than in the non-isolated group (n = 321, 65.2%) (mean = 22.3, *SD* = 5.6 and mean = 17.6, *SD* = 4.6, respectively). Factors associated with high loneliness included individual and family factors (a high number of parenting and life concerns [β = 0.211, *p* < 0.01], not eating breakfast every day [β = 0.087, *p* < 0.05], and fewer partners’ supportive behaviors for household duties and childcare [β =  − 0.240, *p* < 0.001]) and community factors (fewer people to consult about parenting [β =  − 0.104, *p* < 0.01] and low community commitment [β =  − 0.122, *p* < 0.05]) with social isolation as a mediator.

**Conclusion:**

Referral to a counseling organization to alleviate worries about parenting and the creation of a child-rearing environment to enhance the recognition of the community may be considered. These findings could help develop intervention programs for the prevention or alleviation of loneliness experienced by mothers and prevent the associated health risks among mothers and child outcomes.

## Background

Loneliness, which occurs due to a lack of either quantitative or qualitative social relationships, is a subjective, uncomfortable, and painful experience [1, 2]. Loneliness influences the mental health of adult women worldwide, and women constitute a higher risk population than men [3]. Particularly, mothers staying at home with young children have been identified as a high-risk population for loneliness [4, 5]. One previous longitudinal study showed the prevalence of loneliness among mothers was between 34 and 38% [6], and it was associated with both mothers’ depression and children’s adjustment [6, 7]. The stability of mothers’ social and emotional loneliness was high from pregnancy until their child was 18 months old [8, 9]. In particular, mothers with infants and toddlers are at higher risk for loneliness in Japan compared with other OECD countries. This is due to the trend toward nuclear families and the weakening of community ties as well as the fact that fathers are less likely to participate in childcare and mothers have to bear more responsibility and childcare burden [10]. Loneliness among mothers is a social problem worldwide.

It has been noted that loneliness and social isolation are related to each other but should be distinguished [11, 12]. Both concepts may have independent effects on health and, therefore, should be regarded as individual factors, even though both have been associated with a decline in health status and quality of life [13]. The lack of studies highlights the need for mental health programs focused on loneliness, not only adopting an individual approach but also a family and community approach [5] from an ecological systems model [14].

Limited studies have focused on loneliness in mothers with infants and toddlers [9, 15–20]. It has been suggested that loneliness is caused by local environmental factors [15, 16]. The individual factors that contribute to the development of loneliness include personal inadequacies, developmental deficits, and unfulfilling intimate relationships [16] and the parents experience more of these other psychosocial problems [8]. There are few reports of loneliness among mothers raising infants (incidence rates) in Japan, with only a few surveys in each municipality, and few studies clarifying related factors of loneliness [4, 17–20]. Perceptions of interpersonal relationships as well as perceived burden of childcare [17], family [17], childcare supports [17–19], virtual social networks and SNS use [19], and real-world social networks [20] have been identified as factors related to loneliness. However, all these factors are either demographic or social relationship and family-related factors. Associations between loneliness, and individual factors (e.g., behavior) and community factors (e.g., attitude, relationship between community members) have not yet been explored among mothers with young children. Recent studies on loneliness in the general population have reported associations of loneliness with individual variables; health-related behavioral variables [3, 22, 23]; community variables, such as trust in community neighborhood [22], and frequency of neighbor contact among adults [11]; and social capital [24].

To prevent low psychological well-being among mothers with infants and toddlers, it is essential to conduct and evaluate empirical studies that focus on loneliness. The aim of the current study was to identify individual, family, and community factors associated with loneliness in mothers of children less than 3 years of age from an ecological systems model perspective [14] with social isolation as a mediator.

## Methods

### Study design

The study used a cross-sectional design with anonymous, self-report questionnaires.

### Setting

The setting was a community health center in Yokohama City, which is the second largest city in a metropolitan area in Japan.

### Participants

The target population was mothers of infants and toddlers, visiting a community health center for their child’s medical health check-up in Yokohama City, which is the second largest city in Japan, in 2019. Health check-ups, including growth and development examinations and health counseling, are mandatory at 4, 18, and 36 months of age under the Maternal and Child Health Act in Japan. The target population comprised 649 mothers of children aged 4, 18, and 36 months between November 2019 and February 2020. Mothers who could understand Japanese and answer questionnaire items were eligible for participation. This desired sample size was set at one quarter of the number of 0-, 1-, and 3-year-old children per year in this district. Sample size was calculated using G*Power version 3.1.9.7 for Windows [25, 26]. With 95% power, 0.05 level of significance, 0.15 effect size (medium) in multiple regression analysis [27], and 13 predictors, the required sample size for the multiple regression model was calculated to be 189. The minimum  response rate was expected to be 30%. The questionnaires were sent by mail to the 649 mothers along with announcement letters for a health check-up. All eligible mothers were asked to fill the questionnaires and return them by mail.

## Study variables

### Loneliness

The 10-item version of the University of California Los Angeles Loneliness Scale version 3 (The UCLA-LS3-J SF-10 [20]) was used to evaluate loneliness. Each of the 10 items of the UCLA-LS3-J SF-10 [20, 28] has 4 choices: (1) never, (2) rarely, (3) sometimes, and (4) always. The total score ranges from 10 to 40, with higher scores indicating a higher level of loneliness. The reliability and validity of this scale have been established by the authors [20] after the second author (ET) obtained permission to translate the UCLA-LS3-J from its original author (Dr. Russell). The Cronbach’s alpha of the Japanese translated version of the scale was 0.888 [20].

### Social isolation (social network size)

The Japanese version of the Lubben Social Network Scale (LSNS-6 [29, 30]) was used to evaluate social networks. This scale was chosen because it allows for comparisons between the size and quality of social networks. The LSNS-6 comprises 6 items each with 6 response options from 0 (*nobody*) to 5 (*more than 9 persons*) that evaluated the mother’s social network in her family (3 items) and among her friends (3 items). The total score ranges from 0 to 30, with higher scores indicating a larger social network. A score of less than 12 marks the cutoff point for social isolation. Based on the LSNS-6 cut-off value of 12 points, the LSNS-6 was converted to a binary variable where 1 = isolated and 0 = non-isolated. The reliability and validity of the Japanese version have been established [29, 30]. Cronbach’s alpha for the LSNS-6 was 0.82 in the Japanese translated version [29]. This scale is not under license but permission has been obtained for its use.

### Independent variables

Several independent variables were explored as being factors that were potentially related to loneliness from an ecological systems model [14]. These variables were selected based on literature reviews of previous studies [4–9, 15–21] and 3 focused group interviews with 41 professionals or community volunteers, and 31 one-to-one interviews with mothers raising children less than 3 years of age.

*Demographic data,* such as age, family structure (nuclear family; parents and children, extended family, and single-parent family), employment status (housewife, full-time office worker, part-time worker, contract/temporary worker, and self-employed), number of years of residence (less than 1 year, more than 1 year and less than 5 years, more than 5 years and less than 10 years, over 10 years), age of children eligible for health screening, number of children, and employment status of spouse/partner were collected.

*Individual and family factors* comprised of parenting concerns, and the health behaviors of caregivers. The number of parenting concerns was created from 12 dichotomously rated items (1 = *yes* and 0 = *no*), reflecting stressors on parenting and life (e.g., children’s toileting problems, how to play with and pamper their child, financial worries, and balancing work and childcare). The health behaviors of mothers were measured using the Good Health Habits Scale developed by Breslow [31] that were dichotomously rated items (1 = *yes* and 0 = *no*) regarding the respondent’s health behaviors (e.g., sleep, smoking, diet, exercise, alcohol use).

*Partners’ supportive behaviors for household and childcare* were assessed using the partners’ supportive behaviors for household and childcare scale [32]. This scale comprises 29 items that were rated on a 4-point scale from 1 (*do not at all*) to 4 (*do often*) that evaluated the supportive behaviors of the mothers’ partners in terms of emotional support (14 items), and household and childcare (15 items). The scale score is calculated by dividing the sum of the crude scores of all items by the number of items, with higher scores indicating more supportive behaviors by partners. The reliability and validity of this scale have been established [32]. This scale is not under license but permission has been obtained for its use.

*Community factors* comprised of people consulted about parenting, interest in and desire to interact with the neighbors, use of community childcare resources, and recognition of the community (community commitment). The number of people that the mother consulted about parenting was created using 12 dichotomous items (1 = *yes* and 0 = *no*) reflecting parenting social support (e.g., consulted with parents, friends, family doctor, child welfare commissioner). One dichotomous item was used to measure interest in neighbors and one to assess the desire to interact with neighbors. The use of community childcare resources was created from 5 dichotomous items (1 = *yes* and 0 = *no*) that measured resources (e.g., community child rearing support center, comprehensive community support center, volunteer-sponsored park play, parent–child group).

The Community Commitment Scale [33] was used to evaluate recognition of the community. This scale comprises 8 items rated on a 4-point Likert-type scale from 0 (*strongly disagree*) to 3 (*strongly agree*), each evaluating the recognition of the community as an opportunity for socializing (4 items) and belonging (4 items). The total score ranges from 0 to 24, with higher scores indicating a higher community commitment. The reliability and validity of this scale have been established [33]. The Cronbach’s alpha for the CCS was 0.78 in the original scale [33]. This scale is not under license but permission has been obtained for its use.

### Statistical analyses

Based on the LSNS-6 cut-off value of 12 points, the participants were classified into two groups, which were then compared. After calculating the descriptive statistics for each item for all the participants and comparing the two groups using chi-square tests and independent *t*-tests, we analyzed the data for differences in loneliness between the demographic variables using t-tests and one-way analysis of variance (ANOVA). Following the ANOVA, Tukey’s multiple comparisons were performed.

Next, correlations between loneliness and items that had ordinal and continuous scores were examined using Pearson’s correlation coefficients. Among the items with significant correlations (*p* < 0.05), multiple linear regression analyses were performed with loneliness as the dependent variable and the selected items as independent variables, while taking into account multicollinearity with social isolation as a mediator. Age was set as the adjustment variable. The significance level was set at less than 5% on both sides. IBM SPSS version 26 statistical software (SPSS Inc., Chicago, IL, USA) was used to perform all statistical analyses.

## Results

### Respondent characteristics

Out of the total 531 mothers who sent their responses, 492 respondents provided valid responses to all 10 items on the UCLA-LS3-J SF-10 and LSNS-6 (valid response rate: 75.8%).

Table 1 shows the participants’ characteristics. The mother’s social network measured through LSNS-6 had a mean score of 13.3 points (SD: 5.1, range: 0–29), divided into two groups with a cutoff of 12 points, with 171 mothers (34.8%) in the socially isolated group and 321 mothers (65.2%) in the non-isolated group.

**Table 1:** Demographic characteristics (N = 492)

	Total	Low social network (isolated)	High social network (non-isolated)	*p*-value*
	n or (%)	n or (%)	n or (%)	
	Mean ± or SD	Mean ± or SD	Mean ± or SD	
N (%)	492 (100.0)	171 (100.0)	321 (100.0)	
Age (years)	33.2 ± 4.8	33.0 ± 4.8	33.5 ± 5.0	
***Family structure***				
Nuclear family	446 (90.7)	156 (91.2)	290 (90.3)	0.049
Extended family	10 (2.0)	6 (3.5)	26 (8.1)	
Single-parent family	2 (0.4)	8 (4.7)	4 (1.2)	
Others	2 (0.4)	1 (0.6)	1 (0.3)	
***Employment status***				
Housewife	217 (44.1)	78 (45.6)	139 (43.3)	0.710
Full-time office worker	174 (35.4)	61 (35.7)	113 (35.2)	
Part-time worker	14 (2.8)	15 (8.8)	43 (13.4)	
Contract/temporary worker	58 (11.8)	8 (4.7)	12 (3.7)	
Self-employed	20 (4.1)	6 (3.5)	8 (2.5)	
Others	7 (1.4)	2 (1.2)	5 (1.6)	
Missing	2 (0.4)	1 (0.6)	1 (0.3)	
***Number of years of residence***				
< 1 year	54 (11.0)	19 (11.1)	35 (10.9)	0.097
1≦ and < 5 years	220 (44.7)	84 (49.1)	136 (42.4)	
5≦ and < 10 years	99 (20.1)	37 (21.6)	62 (19.3)	
Over 10 years	116 (23.6)	29 (17.0)	87 (27.1)	
Missing	3 (0.6)	2 (1.2)	1 (0.3)	
***Age of children eligible for health screening***				
0 years old	178 (36.2)	69 (40.4)	109 (34.0)	0.542
1 year old	163 (33.1)	51 (29.8)	112 (34.9)	
2 years old	8 (1.6)	3 (1.8)	5 (1.6)	
3 years old	137 (27.8)	47 (27.5)	90 (28.0)	
Missing	6 (1.2)	1 (0.6)	5 (1.6)	
***Number of children***				
One	208 (42.3)	75 (43.9)	133 (41.4)	0.838
Two	218 (44.3)	76 (44.4)	142 (44.2)	
Three	58 (11.8)	18 (10.5)	40 (12.5)	
Four and more	8 (1.6)	2 (1.2)	6 (1.9)	
Missing	0 (0.0)	0 (0.0)	0 (0.0)	
***Employment status of spouse/partner***				
Full-time office worker	436 (88.6)	150 (87.7)	286 (89.1)	0.234
Self-employed	31 (6.3)	10 (5.8)	21 (6.5)	
Contract/temporary worker	6 (1.2)	4 (2.3)	2 (0.6)	
Part-time worker	4 (0.8)	0 (0.0)	4 (1.2)	
House husband	1 (0.2)	1 (0.6)	0 (0.0)	
Others	3 (0.6)	1 (0.6)	2 (0.6)	
Missing	11 (2.2)	5 (2.9)	6 (1.9)	

^*^Chi-square test, t-test or one-way analysis of variance (ANOVA) with Tukey post hoc analysis

There was no difference in the mean age in each group (isolated group: 33.0, SD: 4.8 years; non-isolated group: 33.5, SD: 5.0 years). In terms of family type, there were significantly more single-mother families in the isolated group and three-generation families in the non-isolated group (χ^2^: 9.5, *p* < 0.05). There were no differences in years of residence, age of children eligible for health screening, number of children, the mother’s employment status, or the employment status of spouse/partner.

Table 2 shows loneliness, and individual, family, and community variables. The mean loneliness score of the mothers was 19.2 points (SD: 5.4, range: 10–35, mode: 22). The mean loneliness score was significantly higher in the isolated group than in the non-isolated group; isolated group mean was 22.3 (SD: 5.6, range: 10–32, mode: 22) and non-isolated group mean was 17.6 (SD: 4.6, range: 10–35, mode: 13).

**Table 2:** Loneliness, individual and family, and community variables (N = 492)

	Total	Low	High	*p*-value*
		Social network (isolated)	Social network (non-isolated)	
	n or (%)	n or (%)	n or (%)	
	Mean ± or SD	Mean ± or SD	Mean ± or SD	
N (%)	492 (100.0)	171 (100.0)	321 (100.0)	
***Loneliness***				
UCLA LS3-SF10	19.2 ± 5.4	22.3 ± 5.6	17.6 ± 4.6	< 0.001
***Individual and family factors***				
Number of parenting and life concerns	2.7 ± 2.2	3.0 ± 2.3	2.5 ± 2.2	0.007
Children’s toileting problems	111 (22.6)	49 (28.7)	62 (19.3)	0.017
How to play with and pamper the child	83 (16.9)	37 (21.6)	46 (14.3)	0.037
Financial worries	119 (24.2)	55 (32.2)	64 (19.9)	0.002
Balancing work and childcare	151 (30.7)	64 (37.4)	87 (27.1)	0.017
*Health behaviors of mothers*				
Not sufficient rest from sleep	254 (51.6)	101 (59.1)	153 (47.7)	0.013
No exercise habit	431 (87.6)	146 (85.4)	285 (88.8)	0.439
Smoking	17 (3.5)	5 (2.9)	12 (3.7)	0.646
Not eating breakfast every day	92 (18.7)	32 (18.7)	60 (18.7)	0.972
Drink alcohol every day	28 (5.7)	10 (5.8)	18 (5.6)	0.901
*Partners’ supportive behaviors scale*				
Emotional support	41.8 ± 8.8	41.0 ± 9.0	42.2 ± 8.7	0.187
Household and childcare	45.9 ± 8.7	45.2 ± 8.4	46.2 ± 8.9	0.258
Total partners’ supportive behaviors	87.7 ± 15.2	86.3 ± 15.6	88.4 ± 15.2	0.135
***Community factors***				
Interest in interacting with neighbors				
No	180 (36.6)	84 (49.1)	96 (29.9)	< 0.001
Desire to interact with neighbors				
No	209 (42.5)	92 (53.8)	117 (36.4)	< 0.001
Not greeting their neighbors	23 (4.7)	7 (4.1)	16 (5.0)	0.003
*Number of community childcare resources used*	2.0 ± 1.7	1.8 ± 1.5	2.1 ± 1.8	0.030
Community child rearing support center	236 (48.0)	94 (55.0)	142 (44.2)	0.023
Comprehensive community support center	173 (35.2)	48 (28.1)	125 (38.9)	0.016
Volunteer-sponsored park play	82 (16.7)	17 (9.9)	65 (20.2)	0.003
Parent–child group	72 (14.6)	14 (8.2)	58 (18.1)	0.003
*Number of people to consult about parenting*	3.8 ± 1.5	3.2 ± 1.3	4.1 ± 1.6	< 0.001
Talk to parents	402 (81.7)	125 (73.1)	277 (86.3)	< 0.001
Talk to friends	356 (72.4)	90 (52.6)	266 (82.9)	< 0.001
Consulted with family doctor	241 (49.0)	60 (35.1)	181 (56.4)	< 0.001
*Community commitment scale*	13.0 ± 4.3	11.7 ± 4.1	13.6 ± 4.2	< 0.001
Socializing	6.6 ± 2.6	6.0 ± 2.6	6.9 ± 2.5	< 0.001
Belonging	6.4 ± 2.3	5.7 ± 2.2	6.7 ± 2.2	< 0.001

UCLA LS3-SF10: UCLA loneliness scale ver. 3 short-form 10 items

*Chi-square test, t-test or one-way analysis of variance (ANOVA) with Tukey post hoc analysis

The mothers in the isolated group had a significantly higher total number of parenting concerns than in the non-isolated group (t: 2.72, *p* < 0.05). Significantly more mothers in the isolated group had concerns about play and discipline (χ^2^: 4.37, *p* < 0.05), toileting (χ^2^: 5.74, *p* < 0.05), balancing work and childcare (χ^2^: 5.70, *p* < 0.05), and significantly more mothers had financial concerns (χ^2^: 9.69, *p* < 0.01) than in the non-isolated group. Significantly fewer mothers in the isolated group were well rested by sleep (χ^2^: 6.14, *p* < 0.05) in terms of health behavior. There were no significant differences in fathers’ scores on supportive behavior in the form of domestic work.

In terms of the community factors, mothers in the isolated group were significantly more likely than those in the non-isolated group to "not greet" their neighbors (χ^2^: 13.0, *p* < 0.001). Significantly more mothers in the isolated group were not interested in their neighbors, did not want to interact with them (χ^2^: 18.7, *p* < 0.001), and had significantly lower community commitment scores (socializing and belonging) than those in the non-isolated group (t: − 4.75, *p* < 0.001). Mothers in the isolated group used significantly fewer social resources than those in the non-isolated group (t: − 2.08, *p* < 0.05). The mothers in the isolated group reported significantly lower total number of people consulted about parenting than the mothers in the non-isolated group (t: − 6.38, *p* < 0.05). Significantly lesser mothers in the isolated group than in the non-isolated group, consulted with their parents (χ^2^: 13.0, *p* < 0.05), friends (χ^2^: 52.0, *p* < 0.05), or family doctors (χ^2^: 20.3, *p* < 0.001) about childcare.

### Relationships between the total score of loneliness, demographic variables, and individual, family, and community factors

Table 3 shows the relationships between loneliness, demographic variables, and individual, family, and community factors. The relationships between the total score of loneliness SF-10, and demographic data and individual, family, and community factors were analyzed. In all mothers, significant positive correlations were found between loneliness and the number of parenting and life concerns (r = 0.338, *p* < 0.001), not eating breakfast every day (r = 0.124, *p* < 0.01), not sufficient rest from sleep (r = 0.184, *p* < 0.001), and no exercise habit (r = 0.098, *p* < 0.05). Furthermore, a significant negative correlation was found with partners’ supportive behaviors for household and childcare (r = − 0.360, *p* < 0.001), number of people to consult about parenting (r = − 0.250, *p* < 0.05) and community commitment (r = − 0.301, *p* < 0.001; Table 3).

**Table 3:** Relationship between loneliness and demographics and individual, family, and community factors (N = 492)

		n	Correlation r or Mean ± SD	*p*-value
***Demographic characteristics***				
Age (yrs)		482	0.073	0.107
*Age of children eligible for health screening*				
0 years old		178	19.2 ± 5.5	0.491
1 years old		163	18.9 ± 5.5	
2 years old		8	17.6 ± 5.4	
3 years old		137	19.7 ± 5.3	
*Employment status*				
Housewives		217	19.7 ± 5.7	0.262
Full-time office workers		174	18.4 ± 4.8	
Part-time workers		58	19.2 ± 5.3	
Contract/temporary workers		20	19.5 ± 6.3	
Self-employed		14	20.4 ± 6.2	
Others		7	20.4 ± 5.4	
*Number of years of residence*				
< 1 year		54	19.6 ± 4.8	0.015
1≦ and < 5 years		220	19.9 ± 5.8	
5≦ and < 10 years		99	19.0 ± 5.2	
Over 10 years		116	17.9 ± 5.0	
***Individual and family factors***				
*Number of parenting and life concerns*		491	0.338	< 0.001
Illness and developmental problems	Yes	83	21.2 ± 6.3	0.002
	No	408	18.8 ± 5.1	
Food-related problems	Yes	155	20.1 ± 5.9	0.010
	No	335	18.8 ± 5.1	
Sleep problems	Yes	89	20.4 ± 5.4	0.028
	No	401	19.0 ± 5.4	
How to play with and pamper the child	Yes	83	22.2 ± 5.5	< 0.001
	No	408	18.6 ± 5.2	
Discipline problems	Yes	238	20.2 ± 5.7	< 0.001
	No	253	18.3 ± 5.0	
Children’s toileting problems	Yes	111	20.8 ± 6.2	0.001
	No	380	18.8 ± 5.1	
Financial worries	Yes	119	21.5 ± 5.6	< 0.001
	No	369	18.4 ± 5.1	
Family problems	Yes	100	22.3 ± 5.6	< 0.001
	No	389	18.4 ± 5.0	
Balancing work and childcare	Yes	151	20.4 ± 5.2	0.002
	No	339	18.7 ± 5.4	
*Health behaviors of mothers*				
Not sufficient rest from sleep	Yes	254	20.2 ± 5.7	< 0.001
	No	237	18.2 ± 5.0	
No exercise habit	Yes	431	19.4 ± 5.3	0.031
	No	59	17.8 ± 5.8	
Smoking	Yes	17	21.1 ± 6.8	0.142
	No	474	19.2 ± 5.4	
Not eating breakfast every day	Yes	92	20.6 ± 6.0	0.006
	No	399	18.9 ± 5.2	
Drinking alcohol every day	Yes	28	20.3 ± 5.9	0.268
	No	463	19.2 ± 5.4	
Partners’ supportive behaviors for household duties and childcare		477	− 0.360	<0.001
***Community factors***				
*Number of people to consult about parenting*		492	− 0.250	< 0.001
Consulted with family doctor	Yes	241	18.0 ± 5.0	< 0.001
	No	251	20.4 ± 5.6	
Ask for advice online	Yes	76	20.6 ± 5.9	0.012
	No	416	19.0 ± 5.3	
*Community commitment*		486	− 0.301	< 0.001

Pearson’s coefficient of correlation or one-way analysis of variance (ANOVA) with Tukey post hoc analysis

*p < 0.05

### Factors associated with loneliness (multivariate analysis)

Multiple regression analyses were conducted with loneliness as the dependent variable. Age and number of years of residence were used as adjustment variables. High loneliness was associated with social isolation as a mediator. Individual and family factors associated with high loneliness were: a high number of parenting and life concerns (β = 0.211, *p* < 0.01), not eating breakfast every day (β = 0.087, *p* < 0.05), and fewer partners’ supportive behaviors for household and childcare (β = − 0.240, *p* < 0.001); and the community factor was a fewer number of people to consult about parenting (β = − 0.104, *p* < 0.01), and low community commitment (β = − 0.122, *p* < 0.05). The adjusted R^2^ was 0.376 (Table 4).

**Table 4:** Determinants of loneliness among mothers (n = 492)

Independent variables	β	*p*-value
*Mediating factors: Social isolation (social network size)*		
LSNS-6 (1 = isolated; low social network)	0.310	< 0.001***
***Individual and family factors***		
Number of parenting and life concerns	0.211	< 0.001***
Not eating breakfast every day (1 = yes)	0.087	0.023*
No exercise habit (1 = yes)	0.042	0.258
Not sufficient rest from sleep (1 = yes)	0.041	0.281
Partners’ supportive behaviors for household and childcare	− 0.240	< 0.001***
***Community factors***		
Number of people to consulted about parenting	− 0.104	0.008**
Community commitment	− 0.122	0.002**
Adjusted R^2^	0.376	

Multiple regression analysis (forced entry method): adjusted for age and the number of years of residence

*p < 0.05, **p < 0.01, ***p < 0.001

## Discussion

This study examined the association between loneliness and individual, family, and community factors in mothers raising children less than 3 years of age with social isolation as a mediator. This study arrived at a main result and clarified the factors related to loneliness, including individual and community factors that have not previously been in focus with social isolation as a mediator. As compared to existing studies, this study extends the perspective on loneliness among mothers to individual, family, and community level factors.

The loneliness scores approximated those reported in previous studies [20, 28]; mothers with infants and toddlers in another study conducted in Yokohama city in 2012 [20]: mean: 17.5, SD: 4.9, range: 9–35; and adult men and women working as teachers in the United States [28]: mean: 19.2, SD: 5.1, range: 10–37. Loneliness scores as reported by the mothers with infants and toddlers in this study are similar to those of mothers and other adults, regardless of cultural background.

Our study indicated that higher numbers of parenting concerns were associated with higher loneliness. These included issues with child development, growth, etc., implying that loneliness increased due to challenges in life and marital and family discord. Loneliness is defined as an uncomfortable feeling because of the quantitative and qualitative deficiencies in the individual’s social network [1]. Mothers who feel higher levels of loneliness compared to those who feel lower levels of loneliness may not perceive raising their children well or may feel that they are not receiving sufficient social support. This result suggests that health professionals need to consider the parenting concerns of mothers to alleviate their loneliness. In particular, the isolated group with higher feelings of loneliness and more worries may be a target population, because they have less interest in and interactions with their neighbors, have fewer people to consult with regarding parenting, and use fewer social resources than the non-isolated mothers.

Consistent with a recent study among university students conducted in 28 countries [34], this study found that skipping breakfast and not eating breakfast every day, were associated with mothers’ loneliness. Previous research proposed that breakfast contributes to the production of serotonin, which regulates depression, irritability, and cognitive function [35]. However, the pathways through which skipping breakfast is associated with loneliness are unclear among mothers. Further research on the association between skipping breakfast and loneliness among mothers of infants and toddlers is needed.

Similar to one previous study in Japan [18], we found that loneliness is associated with partner’s parenting and housework support behavior. More childcare support in the family can alleviate the mother’s sense of childcare burden and help reduce loneliness [17]. This may particularly be a problem in Japan, needing to be addressed as a society. The average time spent by husbands on housework and childrearing in Japan is 83 min per day, which is much less than that spent by wives, and remains the lowest among the developed countries [36]. In the fiscal year 2018, the percentage of men taking parental leave was 6.2 percent compared to 82.2 percent for women. Therefore, it is important to disseminate information that will help fathers understand the importance of providing emotional support to mothers [36].

The number of people to consult about parenting was associated with loneliness in mothers. These people could be seen as emotional and/or informational support resources. Social factors, such as opportunities for contact, social support, and social networks have been described as relevant factors to reduce mothers’ loneliness in previous studies conducted in Japan [17, 18]. A systematic review reported an effect of social interaction intervention on loneliness among older adults [37]. A plausible reason is that mothers with more people to consult and more such support factors tend to experience more positive feelings regarding social support and lower feelings of loneliness than mothers with less support of this type.

Furthermore, this study found a significant relationship between community commitment perception of “belonging” and “socializing” and lower feelings of loneliness, among those with a high social network. Regionally associated indices such as social capital [24] and cohesive community [12] have become important factors for residents of all ages in the recent years. A previous study in Finland reported that low levels of trust were associated with loneliness across the 15 to 80 years age group. Social capital in terms of neighborhood cohesion may be of more importance for the prevention of loneliness in younger people [24]. A sense of belonging to the neighborhood may foster neighborhood attachment, or the degree of membership in various neighborhood networks [24]. Previous studies of mothers showed that group participation increased the number of helpful friends [38], allowed mothers to connect with each other [39], and increased social capital and mental wellbeing [15]. Further, high social network size is associated with being connected to the community. The results showed all the items to be significantly related to loneliness, suggesting that involvement in the community and neighborhood would be effective in the prevention of loneliness. For mothers, creating opportunities to interact with each other and creating a child-rearing environment in cooperation with the neighbors can increase community commitment.

Our study has some limitations. First, the respondents in this study were all from a city in a metropolitan area of Japan, even though the response rate was relatively high. It would be beneficial to explore the factors related to loneliness among mothers in a more diverse population, such as mothers in other communities and those who are at a particularly high risk for loneliness. Second, this study used a cross-sectional design and did not show causal relationships. Further longitudinal research is needed to clarify the causal relationships among the variables studied in this research. Third, this study analyzed self-reported data; thus, there is an issue with shared method variance. Using objective data from multiple informants and sources in future research would be beneficial to clarify the factors related to loneliness. Finally, the model and factors in this study may not have covered all the factors related to loneliness among mothers because the adjusted R^2^ showing explanatory rate was relatively low. Further empirical research is needed to explore the other factors. One of the major strengths of this study is that we extended the range of the factors associated with loneliness and clarified individual factors such as worries, lifestyle behaviors, and community commitments as community factors, which have not previously been focused upon. We used a standardized tool for defining loneliness and social isolation using a reliable and validated scale, distinguishing between social isolation and loneliness. The findings of this study may contribute to the elucidation of the mechanism underlying loneliness in mothers with infants and toddlers, and ultimately to the development of programs to alleviate the same.

## Conclusion

This study extended the range of the factors associated with loneliness and clarified individual factors and community factors that have not previously been focused upon when studying loneliness among mothers. Our study indicates that public health professionals could focus on individual factors and community factors and offer a variety of approaches, rather than a one-size-fits-all approach when addressing loneliness in mothers of small children. It would be beneficial for mothers to receive referrals to counseling organizations that focus on parenting concerns (e.g., individual factors), and to have access to child-rearing environments that enhance the recognition of community-level benefits. Future research needs to examine the mechanisms that might the influence of individual, family, and community factors explored in this study.

## Data Availability

The datasets generated and analyzed during the current study are not publicly available because the Ethical Guidelines for Epidemiological Research by the Japanese Government, and the National Basic Resident Registration System administered by the Ministry of Internal Affairs and Communications in Japan prohibit researchers from providing their research data to third-party individuals.

## Abbreviations

UCLA-LS3-J: Japanese version of the University of California Los Angeles Loneliness Scale version 3
SF-10: Ten-item version of the University of California Los Angeles Loneliness Scale version 3
SD: Standard deviation
LSNS-6: The Lubben Social Network Scale
